# Efficacy and cultural appropriateness of psychosocial interventions for paediatric burn patients and caregivers: a systematic review

**DOI:** 10.1186/s12889-020-8366-9

**Published:** 2020-03-04

**Authors:** H. M. Williams, K. Hunter, K. Clapham, C. Ryder, R. Kimble, B. Griffin

**Affiliations:** 1 Centre for Children’s Burns and Trauma Research, Child Health Research Centre, The University of Queensland, Graham Street, South Brisbane, 4101 QLD Australia; 2 Pegg Leditschke Paediatric Burns Centre, Queensland Children’s Hospital, Graham Street, South Brisbane, QLD 4101 Australia; 3 Injury Division, The George Institute for Global Health, University of New South Wales, King Street, Sydney, 2042 NSW Australia; 4 Ngarruwan Ngadju First Peoples Health and Wellbeing Research Centre, Australian Health Services Research Institute, The University of Wollongong, Wollongong, 2522 NSW Australia; 5 College of Medicine & Public Health, Southgate Institute for Health Society and Equity, Flinders University, Registry Road, Bedford Park, 5042 SA Australia; 6 School of Nursing, Queensland University of Technology, Ring Road, Brisbane, 4059 QLD Australia

**Keywords:** Aboriginal, Torres Strait Islander, Paediatric burns, Psychosocial interventions, Pain, Anxiety, Distress, Psychological trauma

## Abstract

**Background:**

Paediatric burns are highly painful and traumatising injuries that are overrepresented among Aboriginal and Torres Strait Islander people. Paediatric burn patients’ pain remains poorly managed by pharmacological interventions, leading to increased anxiety, distress, and trauma in patients and their caregivers. Non-pharmacological psychosocial interventions have been suggested as effective in reducing pain and psychological morbidities among paediatric burn patients and their caregivers; however, their degree of effectiveness and appropriateness for Aboriginal and Torres Strait Islander people is unclear.

**Methods:**

A non-date restricted systematic review was conducted through four databases. Studies published in English assessing psychosocial interventions on paediatric burn patients’ physical pain along with theirs and/or their caregiver’s anxiety, distress, or trauma symptoms were identified and included in this review. Included studies were assessed for their ability to reduce one of the outcomes of interests and for their reflection of Aboriginal and Torres Strait Islander peoples’ perspectives of health.

**Results:**

Of the 3178 identified references, 17 were eligible. These include distraction based techniques (*n* = 8), hypnosis/familiar imagery (*n* = 2), therapeutic approaches (*n* = 4), and patient preparation/procedural control (*n* = 3). Distraction techniques incorporating procedural preparation reduced pain, while discharge preparation and increased ‘patient control’ reduced patient and caregiver anxiety; and internet based Cognitive Behaviour Therapy reduced short-term but not long-term post-traumatic stress symptoms. No interventions reflected Aboriginal and Torres Strait Islander peoples’ perspectives of health; and few targeted caregivers or focused on reducing their symptoms.

**Conclusions:**

The development and assessment of psychosocial interventions to appropriately meet the needs of Aboriginal and Torres Strait Islander paediatric burn patients is required.

## Background

Burn injuries cause severe pain [1–5] and can result in psychological trauma [2–4], anxiety [3, 5] and distress [1, 3]. These uniquely challenging injuries affect Aboriginal and Torres Strait Islander people at higher rates than non-Indigenous Australian people. This is highlighted by the Burns Registry of Australia and New Zealand’s most recent report that between 2017 and 2018 Aboriginal and Torres Strait Islander people were hospitalised for burn injuries three times more often than non-Indigenous people, and experienced significantly larger burns covering 10–49% of their Total Body Surface Area (TBSA) [6]. Paediatric specific data indicates similar discrepancies with Aboriginal and Torres Strait Islander children and adolescents experiencing 2.4 times higher rate of hospitalisation from burn injuries than non-Indigenous Australian children between 2011 and 2013 [7].

A unique challenge of these injuries lies within the persistent and debilitating base level of pain that is further intensified by regular procedures undergone for months to years following the initial injury [8, 9]. The complex nature of burn related pain often results in poor management despite the administration of standard doses of analgesia [8–13] and is particularly difficult to monitor among paediatric burn patients who are less able to articulate the intensity of their own pain [14]. This is further complicated for Aboriginal and Torres Strait Islander people who may not report their pain at all [15, 16] or verbally express their pain differently to non-Indigenous Australians [17]. This is particularly concerning as poorly managed pain during hospitalisation strongly predicts burn patients’ psychosocial adjustment and overall wellbeing up to two years following treatment and hospital discharge [18].

More specifically, the pain and discomfort experienced by burn patients is associated with increased distress and anxiety [19]. This, in turn, increases the risk of developing other psychological morbidities such as acute stress and post-traumatic stress disorders (PTSD) [19]. The impact of burn related pain and distress is further exacerbated for paediatric burn patients who have a limited understanding of their injury and treatment [20], restricted agency in their care [21], and reduced ability to cope with the unpredictability of a hospital setting [22]. The struggles faced by paediatric burn patients is also greatly felt by their caregivers [20] who often experience overwhelming feelings of guilt, worry, panic, and anxiety whilst struggling with drastic shifts in their parenting role and ability to assist their child [23]. A review of empirical data highlights that 10–20% of paediatric burn patients and 4–42% of their caregivers reportedly experience symptoms of PTSD following the burn injury [24]. This highlights the need to effectively treat paediatric burn patients’ and their caregivers’ anxiety, distress, and psychological trauma. This is further emphasised by the finding that early onset of such psychological morbidities have a high rate of relapsing later in life [25].

The use of non-pharmacological, psychosocial interventions in conjunction with pharmacological analgesia have been suggested for reducing burn related pain and consequential psychological morbidities [26]. Interventions incorporating Gate Control Theory [21] techniques into change of dressing (COD) procedures are suggested as particularly effective in distracting the patient and reducing their ability to concentrate on painful stimuli [27]. Following this theory, virtual reality has shown particularly favourable results on pain management among paediatric burn patients alone [28], and combined with young adult burn patients [29, 30]. Likewise, music therapy has shown promising effects in reducing anxiety and distress among paediatric burn patients alone [31–33], and combined with adults [34, 35]. Other psychosocial interventions utilising cognitive approaches and behavioural strategies have demonstrated similar effects and suitability for use among a wide age range of children and adolescents. More specifically, cognitive approaches including imagery, preparation techniques, information sharing, and coping strategies are suggested as particularly suitable for older children and adolescents [14]. While behavioural strategies including breathing exercises, desensitisation, and positive reinforcement are suggested as particularly suitable for younger children [14].

Several studies have presented the usefulness of such psychosocial interventions, however, no comprehensive comparison or systematic review has been conducted to assess their rigour, effectiveness, or appropriateness in meeting the needs of Aboriginal and Torres Strait Islander paediatric burn patients and their caregivers. This study assessed the effectiveness of any psychosocial intervention in reducing pain and psychological trauma, distress, and/or anxiety among paediatric burn patients and their caregivers generally. Alongside this assessment, we systematically evaluated the appropriateness and applicability of such interventions for use among Aboriginal and Torres Strait Islander families to inform the necessity and directions for future developments of culturally appropriate interventions.

## Methods

### Protocol and registration

Details of the protocol for this systematic review were registered on PROSPERO, the international prospective register of systematic reviews (CRD42018073451) [36].

### Eligibility criteria

The below eligibility criteria were applied (Table 1). Associated search terms were developed in consultation with experts from the University of Queensland library (see Additional file 1).

**Table 1:** Eligibility criteria

Inclusion	Exclusion
1. Studies focused on unintentional pediatric burn injuries. 2. Injured children < 18 years receiving treatment at time of study, and/or their caregivers. 3. Assessment of psychosocial interventions^a^ 4. Randomised control trials (RCT) or non-randomised control trials (NRCT) with clear comparison groups. 5. Assessing patient pain and theirs and/or caregiver’s anxiety, distress, and/or trauma symptoms. 6. Studies published in English with no date restrictions.	1. Focus on non-burn injuries/illnesses or intentional burn injuries. 2. Injured adults > 18 years and/or injured children < 18 years post burns care. 3. Assessment of physical interventions i.e. dressings, physical therapy, massage etc. 4. Studies with no clear comparison group. 5. Assessment of any other outcome variable, or studies measuring only pain.

^a^Defined here as any intervention designed primarily to improve psychosocial wellbeing rather than physiological aspects of health

### Information sources

The Cumulative Index to Nursing and Allied Health Literature (CINAHL), Embase, MEDLINE, PubMed, and PsycINFO databases were systematically searched up to November 2019. Database alerts were established at this time and resulting references added as they became available. Reference lists of included manuscripts were hand-screened to identify additional articles not previously captured.

### Study selection

Duplicates were removed prior to the lead author double screening all title and abstract references in the online systematic review software package Covidence, Australia and Argentina (v1086 e0dda871) [37]. An additional reviewer screened 10% of title and abstract references in Excel to verify accuracy with 91% agreeance. The lead author screened all full text references and two additional authors screened 50% each of full text references. Conflicts were resolved via consensus among all authors.

### Assessment of cultural components, study quality and risk of bias

Cultural components were extracted via a form developed in line with Milroy et al.’s the *Dance of Life* [38], a multi-dimensional model reflecting the interconnectedness and complex layering of Australian Aboriginal people’s perspectives of health and wellbeing. In accordance with the model, data was extracted on five core aspects of health: physical (four items), psychological (three items), social (four items), spiritual (three items), and cultural wellbeing (three items). All items were graded zero (not present) or one (present) with relevant details extracted. The cultural components data form is included as supplementary materials (see Additional file 2).

Risk of bias was assessed against the *Cochrane Collaboration’s tool for assessing risk of bias* [39] (nine items), and an additional three items from the *Cochrane suggested risk of bias criteria for EPOC reviews* [40] to assess potential intervention contamination, and baseline outcome and characteristic similarities. All risk of bias items were graded zero (high/unclear bias) or one (low risk of bias). Study quality was evaluated by the *Downs & Black Checklist* [41] (28 items) to assess reporting, external validity, internal validity, and study power. Power was graded based on the smallest sample group (0 = *n*_< 1_, 1 = n_1_-n_2_, 2 = n_3_-n_4_, 3 = n_5_-n_6_, 4 = n_7_-n_8_, 5 = *n*_> 8_), and all other items graded zero (no) or one (yes); possible maximum score of 31. Hooper et al.’s classifications [42] were adapted to provide overall quality rankings of poor (≤14), fair (15–19), good (20–25), or excellent (26–31). Assessment and data extraction were conducted by the lead author for all studies, and independently by two co-authors for 50% of studies.

### Data synthesis

Meta-analysis was not appropriate due to high heterogeneity among studies. Therefore, data was synthesised narratively, presented in text by outcome (i.e. pain, anxiety, distress, and trauma), and tabulated by intervention type.

## Results

### Study selection

**Fig. 1:**
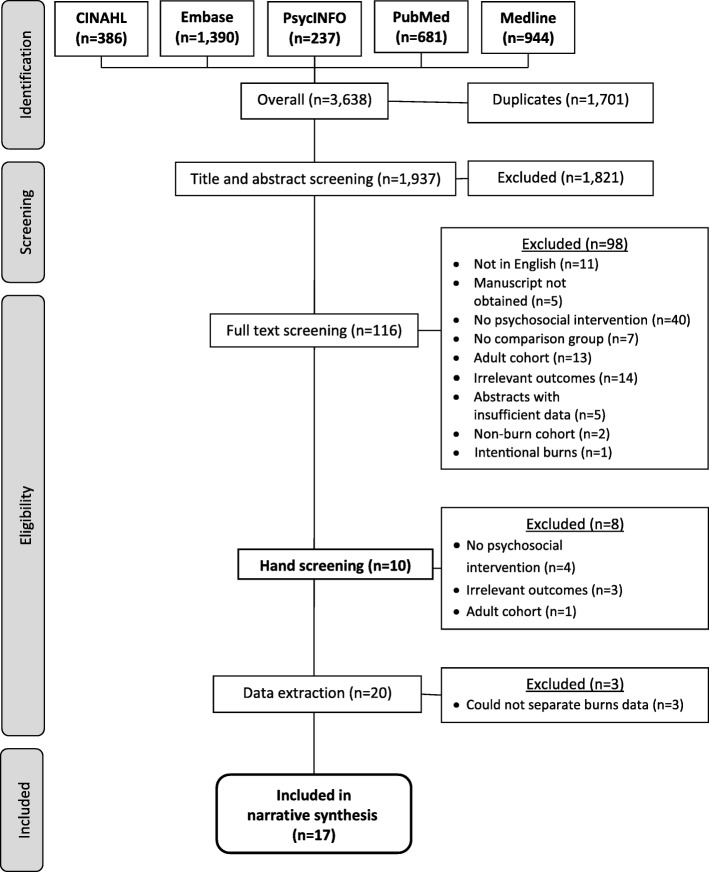
PRISMA flow chart of inclusion/exclusion rates

Database searches returned 3638 abstracts published prior to 18th November 2019. Duplicates were removed and the remaining 1937 abstracts underwent title and abstract screening, resulting in 1821 exclusions and 116 inclusions for full text revision. Exhaustive attempts to obtain full manuscripts was successful for 100 abstracts. Of the remaining 16, five were not able to be acquired and 11 were unavailable in English. Full manuscripts were double screened by the lead author and two co-authors, resulting in a further 82 exclusions. References of the remaining 18 studies were hand-screened, identifying an additional ten abstracts of interest, two of which were included for data extraction (see Additional file 3). Data extraction was not possible for three studies despite thorough attempts to contact study authors to obtain missing data and further information. Data was extracted from the final 17 eligible studies and narratively synthesised. Exclusion rates are outlined in the PRISMA flow diagram (Fig. 1).

### Main results

The participant and study characteristics of included interventions are presented in Tables 2 and 3 respectively. The key findings of included interventions are presented by intervention type in Table 4, and narratively synthesised by outcome below.

**Table 2:** Participant characteristics

Reference	Group	N	Age M (SD)	Age Range	Male n (%)	TBSA% M (SD)	Ethnicity n (%)		
							Reported	Not reported	First Nation
Blakeney 2005 [43].	I	32	14 (1.8)	12–17	9 (28)	36.8 (25.1)	32 (100)	0 (0)	2 (6)^*^
	C	32	14.2 (1.9)		17 (53)	44.2 (20.6)	32 (100)	0 (0)	0 (0)^*^
Brown 2014 [44].	I	47	8.3 (2.5)	4–13	27 (57.5)	1.9 (2.2)	47 (100)	0 (0)	0 (0)
	C	52	8.2 (2.7)		33 (63.5)	1.9 (2.1)	52 (100)	0 (0)	0 (0)
Burns-Nader 2017 [45].	I	15	7.8 (2.3)	4–12	8 (53)	9.2 (10.3)	15 (100)	0 (0)	0 (0)
	C	15	7.1 (2.8)		11 (73)	6.4 (7.5)	15 (100)	0 (0)	0 (0)
Chester 2018 [46].	I	29	8.6 (3.4)	4–15	16 (59)	1.2 (2.0)^^^	0 (0)	29 (100)	0 (0)^**^
	C	35	7.1 (2.7)	4–15	22 (63)	1.0 (2.0)^^^	0 (0)	35 (100)	0 (0)^**^
Elliott 1983 [47]	I	4	8.5 (3.5)	5–12	4 (100)	21.5 (15.0)	3 (75)	1 (25)	0 (0)
	C	4	6.7 (2.1)	5–9	4 (100)	32 (24.3)	1 (25)	3 (75)	0 (0)
Foertsch 1998 [48]	I	13	5.8	3–12	12 (52)	11.4	0 (0)	13 (100)	0 (0)
	C	10					0 (0)	10 (100)	0 (0)
Hyland 2015 [49]	I	50	2.3 (1.5–4.5)^^^	0–16	25 (50)	0.8 (0.5–2.0)^^^	0 (0)	50 (100)	0 (0)
	C	50	2.2 (1.6–3.9)^^^		27 (54)	0.5 (0.5–2.0)^^^	0 (0)	50 (100)	0 (0)
Jeffs 2014 [50].	I^VR^	8	14.3 (2.0)	10–17	3 (38)	7.4 (8.5)	6 (75)	2 (25)	0 (0)
	I^PD^	10	12.6 (2.1)		8 (80)	3.4 (3.3)	9 (90)	1 (10)	0 (0)
	C	10	13.9 (2.8)		8 (80)	4.7 (6.9)	10 (100)	0 (0)	0 (0)
Kavanagh 1983 [51]	I	4	6.3 (4.4)	2–11	4 (100)	22.7 (9.1)	0 (0)	4 (100)	0 (0)
	C	5	7.1 (3.8)	2.5–11.5	3 (60)	37.5 (27.1)	0 (0)	5 (100)	0 (0)
Kipping 2012 [52]	I	20	12.6 (1.3)	11–17	13 (65)	5.1 (6.3)	0 (0)	20 (100)	0 (0)
	C	21	13.5 (1.8)		15 (71)	4.7 (4.5)	0 (0)	21 (100)	0 (0)
Miller 2010 [53]	I^VGD^	20	6.6 (2.5)	3–10	12 (60)	2.6 (1.4)	0 (0)	20 (100)	0 (0)
	I^MMD-D^	20	6.6 (2.6)		13 (65)	2.8 (1.9)	0 (0)	20 (100)	0 (0)
	I^MMD-PP^	20	5.5 (2.1)		14 (70)	4.3 (4.2)	0 (0)	20 (100)	0 (0)
	C	20	6.1 (2.1)		8 (40)	2.5 (1.4)	0 (0)	20 (100)	0 (0)
Miller 2011 [54]	I	20	6.0 (2.0)	3–10	12 (60)	2.8 (1.0)	20 (100)	0 (0)	0 (0)
	C	20	5.9 (2.5)		9 (45)	2.2 (1.1)	20 (100)	0 (0)	0 (0)
Moore 2015 [55]	I^Patient^	12	3.0^^^	3–6	6 (50)	–	12 (100)	0 (0)	0 (0)
	C^Patient^	9	3.0^^^	3–5	3 (33)	–	9 (100)	0 (0)	0 (0)
	I^Caregiver^	12	34^^^	20–44	1 (8)	N/A	0 (0)	12 (100)	0 (0)
	C^Caregiver^	9	28^^^	23–54	1 (11)	N/A	0 (0)	9 (100)	0 (0)
Quay 1983 [56]	I	26	5.3	0.7–15	–	23 (1–81)	0 (0)	26 (100)	0 (0)
	C	24					0 (0)	24 (100)	0 (0)
Sveen 2017 [57]	I^Patient^	26	5.3 (3.5)	–	13 (50)	8.5 (7.0)	0 (0)	26 (100	0 (0)
	C^Patient^	23	6.4 (3.8)		14 (61)	9.9 (7.0)	0 (0)	23 (100)	0 (0)
	I^Caregivers^	31	36.4 (6.6)	–	9 (29)	N/A	0 (0)	31 (100)	0 (0)
	C^Caregivers^	31	38.3 (5.5)		11 (35)	N/A	0 (0)	31 (100)	0 (0)
Van der Heijden 2018 [58]	I	71	2.0 (13.1–4.1)^^^	0–13	37 (52)	7 (4–13)^	0 (0)	71 (100)	0 (0)
	C	64	1.7 (1.3–2.9)^^^		32 (50)	10 (5–15)^	0 (0)	64 (100)	0 (0)
Whitehead-Pleaux 2006 [59]	I	8	–	6–16	5 (36)	–	0 (0)	8 (100)	0 (0)
	C	6					0 (0)	6 (100)	0 (0)

*I* Intervention, *I*^*VR*^ Virtual reality intervention, *I*^*PD*^ Passive distraction intervention, *I*^*VGD*^ Video game distraction intervention, *I*^*MMD-D*^ Multi-modal Device-Distraction intervention, *I*^*MMD-PP*^ Multi-modal Device-Procedural Preparation intervention, *C* Control, *M* Mean, *SD* Standard deviation, ^Median (IQR), *Native American, **Aboriginal and Torres Strait Islander/South Sea Islander

**Table 3:** Study characteristics

First author, year [reference]	Study design, location	Intervention	Control	Outcome: measures^(assessor*)^	Measurement time points
Blakeney 2005 [43].	RCT, USA.	*n* = 32^^3 (9)^ 4-day group social skills workshop based on Changing Faces REACH OUT, and ‘usual’ treatment.	*n* = 32^^10 (31)^ ‘Usual’ treatment, and follow-up psychological appointments upon request only.	Anxiety/depression: *CBCL*^(C)^.	Pre-intervention and 1 year post-intervention: *CBCL*.
Brown 2014 [44].	RCT, Australia.	*n* = 47^^12 (23)^ Ditto™ PP pre-COD, and distraction interactive story/game during COD.	*n* = 52^^12 (26)^ Standard distraction during COD: TV, videos, books, toys, and caregiver soothing.	Pain: *FPS-R*^(Pt.)^, *HR*^(N)^. Pain and distress: *FLACC*^(N)^. Anxiety: *VAS-A*^(Pt. > 8 yrs)^. Trauma: *CTSQ*^(Pt. > 6 yrs)^.	Pre-randomisation: *FPS-R*, *HR*, *FLACC*, *VAS-A*. Pre-removal: *FPS-R*, *FLACC*, *VAS-A*. Post-removal: *FPS-R*, *HR*, *FLACC*, *VAS-A*, *CTSQ*^(1st COD)^. Post- application: *FPS-R*, *HR*, *FLACC*, *VAS-A*. During removal and application: *HR.* 3mths post re-epithelisation: *CTSQ.*
Burns-Nader 2017 [45].	RCT, USA.	*n* = 15^^0 (0)^ Tablet distraction game, and CLT support during 2nd and/or 3rd COD.	*n* = 15^^0 (0)^ Standard distraction, and CLT support during 2nd and/or 3rd COD.	Pain: *FACES*^(Pt.)^, *nurse’s pain reports*^(N)^. Anxiety: *CEMS*^(Pt.)^.	Prior and during hydrotherapy: *CEMS*. Post-hydrotherapy: *FACEs, nurse’s pain reports*, *CEMS*.
Chester 2018 [46].	RCT, Australia.	*n* = 29^^0 (0)^ Hypnosis pre and during COD: guided imagery, breathing, muscle relaxation, and permissive and direct hypnotic suggestions.	*n* = 35^^0 (0)^ Standard interventions pre and during COD: parent presence, books, TV, electronic games, DVDs, toys, bubbles, music, and Ditto™ PP and distraction.	Pain: *FPS-R*^(Pt.)^, *FLACC*^(N)^, *NRS*^(C)^, *HR*^(R)^. Anxiety: *VAS-A*^(Pt. ≥8 yrs, C < 8 yrs)^. Trauma: *CPSS*^(Pt. > 7 yrs)^, *YCPC*^(C < 7 yrs)^.	Pre and post-procedure: *FPS-R, FLACC, NRS, VAS-A.* During procedure: *FPS-R, FLACC, NRS.* Pre-medication and post-application: *HR.* 3-months post-injury: *CPSS, YCPC.*
Elliott 1983 [47].	NRCT, USA.	*n* = 4^^0 (0)^ Stress management during COD: distraction, breathing, emotive imagery, and pain reinterpretation.	*n* = 4^^0 (0)^ SC during COD.	Pain and distress: *BTDS*^(MS)^.	Removal, first 15mins of hydrotherapy, and during physical therapy and dressing re-application: *BTDS*.
Foertsch 1998 [48].	RCT, USA.	*n* = 13^^Total 1 (43)^ Familiar imagery during COD: focus on childhood memory/experience.	*n* = 10^^Total 1 (43)^ Social support during COD: researcher conversation and encouragement.	Pain and anxiety: *FACES*^(Pt.’s 3–9 yrs)^, *VAS*^(Pt.’s 9–12 yrs)^. Distress: *OSBD*^(R)^.	Baseline and 15-s intervals during procedure: *OSBD.* Post-procedure: *FACES, VAS.*
Hyland 2015 [49].	RCT, Australia.	*n* = 50^^0 (0)^ CLT PP, caregiver support and education, and distraction during COD.	*n* = 50^^0 (0)^ SC, and minimal distraction during COD: caregiver, music, and toys/electronic devises.	Pain: *CHEOPS*^(IA)^, *FACES*^(Pt.’s 5–10 yrs)^, *VAS*^(Pt.’s > 10 yrs)^. Pain and anxiety: *Procedural pain and anxiety questionnaire*^(C)^, *nursing staff questionnaire*^(N)^. Anxiety: *CFS*^(IA, Pt.)^.	Pre-procedure: *Procedural pain and anxiety questionnaire.* 2-min intervals during procedure: *CHEOPS, CFS.* Post-procedure: *FACES, VAS, Procedural pain and anxiety questionnaire, CFS.*
Jeffs 2014 [50].	RCT, USA.	*n* = 8^^0 (0)^ I^VR^: 3D interactive program pre and during COD.	*n* = 10^^0 (0)^ ‘Typical’ care during COD: standard nurse communication.	Pain: *APPT-WGRS*^(Pt.’s 8–17 yrs)^. Anxiety: *STAI-CH*^(Pt.)^.	Pre-procedure: *APPT-WGRS, STAI-CH*.
		*n* = 10^^0 (0)^ I^PD^: PD (i.e. movie) pre and during COD.			
Kavanagh, 1983 [51].	NRCT, USA.	*n* = 4^^0 (0)^ Max. procedure ‘predictability’: specific nurse attire, and ‘patient control’ of ‘appropriate’ aspects of procedure.	*n* = 5^^0 (0)^ Min. ‘predictability’, and medical staff control over procedure.	Pain: *Nurse reports* 0–6 scale^(N)^. Anxiety: *CBI*^(N, C of pt.’s 1–3 yrs)^.	2–3 times daily: *Nurse reports.* Weekly: *CBI.*
Kipping 2012 [52].	RCT, Australia.	*n* = 20^^0 (0)^ Off-the-shelf VR pre and during COD.	*n* = 21^^0 (0)^ Standard distraction during COD: TV, stories, music, and caregiver.	Pain: *VAS*^(Pt., C)^. Pain and distress: *FLACC*^(N)^.	Baseline, and retrospective post-removal and application: *VAS, FLACC.*
Miller 2010 [53].	RCT, Australia.	*n* = 20^^2 (10)^ I^MMD-PP^: MMD-PP pre-COD, and standard distraction during COD.	*n* = 20^^3 (15)^ Standard distraction during COD: toys, TV, and nurse/caregiver interactions.	Pain: *FACES*^(Pt.)^, *VAS*^(C)^. Pain and distress: *FLACC*^(N)^.	Pre and post-removal, pre and post-application: *FACES, VAS, FLACC.*
		*n* = 20^^1 (5)^ I^MMD-D^: MMD-D interactive story/game during COD.			
		*n* = 20^^4 (20)^ I^VGD^: VGD during COD.			
Miller 2011 [54].	RCT, Australia.	*n* = 20^^0 (0)^ MMD-PP, MMD-D interactive story/game pre and during COD.	*n* = 20^^0 (0)^ Standard PP and distraction pre and during COD.	Pain: *FACES*^(Pt.)^, *VAS*^(C)^, *HR*^(N)^. Pain and distress: *FLACC*^(N)^.	Pre and post-removal, pre and post-application: *FACES, VAS, FLACC.* During removal and application: *HR*.
Moore 2015 [55].	NRCT, USA.	*n* = 12^^0 (0)^ CLT MP pre-COD: standard medical equipment, and puppets.	*n* = 9^^0 (0)^ SC during COD: standard PP, and clinical staff verbal explanations.	Pain: *FPS*^(Pt.)^. Pain and distress: *FLACC*^(R)^. Anxiety: *STAI-CH*^(C)^.	Pre and post-procedure: *FPS, FLACC, STAI-CH.* Post-removal: *FPS.*
Quay 1983 [56].	RCT, USA.	*n* = 26^^0 (0)^ Discharge preparation weekly by nurse, written information, and procedural rehearsal 3 days pre-discharge.	*n* = 24^^0 (0)^ Routine instructions 3 days pre-discharge.	Anxiety: *STAI-CH*^(Pt., C)^.	1-day pre-discharge, 1st follow-up visit: *STAI-CH.*
Sveen 2017 [57].	RCT, Sweden.	*n* = 31^^16 (52)^ Internet based CBT and ACT support program.	*n* = 31^^3 (10)^ SC during COD.	Post-traumatic stress: *IES-R*^(*C*)^, *PSI-SF*^(*C*)^, *PSS*^(C)^.	Pre-procedure, post-procedure, 3mths post-injury, 12mths post-injury: *IES-R*, *PSI-SF*, *PSS.*
Van der Heijden 2018 [58].	RCT, South Africa.	*n* = 71^^3 (4)^ 3–5 min MT, and parental soothing post-COD.	*n* = 64^^3 (4)^ SC during COD, and parental soothing post-COD.	Pain: *COMFORT-B*^(R)^, *FACES*^(Pt.)^. Distress: *OSBD-r*^(R)^, *FPS-R*^(Pt.)^.	Pre-procedure, hallway, entering room: *OSBD-r, COMFORT-B.* Pre and post-procedure: *FPS-R, FACES.*
Whitehead-Pleaux 2006 [59].	RCT, USA.	*n* = 8^^0 (0)^ MT during COD.	*n* = 6^^0 (0)^ Verbal support and distraction by music therapists during COD.	Pain: *FACES*^(Pt.)^, *HR*^(R)^. Behavioral distress: *NAPI*^(R)^. Anxiety: *Fear Thermometer*^(Pt.)^.	Pre and post-procedure: *FACES, HR, Fear Thermometer.* During procedure: *FACES, HR, NAPI, Fear Thermometer.*

*Assessors: *Pt* patient, *C* caregiver, *N* nurse, *R* researcher, *IA* independent assessor, *MS* medical student. *^*Attrition rate n (%). *ACT* Acceptance and Commitment Therapy, *APPT-WGRS* Adolescent Paediatric Pain Tool, Word Graphic Rating Scale, *BTDS* Burn-Treatment Distress Scale, *CBCL* Children’s Behavior Checklist, *CBI* Children’s Behavior Inventory, *CBT* Cognitive Behavioral Therapy, *CEMS* Children’s Emotional Manifestation Scale, *CFS* Children’s Fear Scale, *CHEOPS* Children’s Hospital of Eastern Ontario Pain Scale, *CLT* Child Life Therapy, *COD* Change of dressing, *COMFORT-B* COMFORT-Behavioral scale, *CPSS* Child PTSD Symptom Scale, *CTSQ* Child Trauma Screening Questionnaire, *FACES* Wong-Baker FACES pain rating scale, *FLACC* Faces Legs Arms Cry Consolability, *FPS* Faces Pain Scale, *FPS-R* Faces Pain Scale-Revised, *HR* Heart rate, *IES-R* Impact of Event Scale-Revised, *MMD* Multi-modal Device, *MMD-D* Multi-modal Device-Distraction, *MMD-PP* Multi-modal Device-Procedural Preparation, *MP* Medical play, *MT* Music therapy, *NAPI* Nursing Assessment of Pain Index, *NRS* Numeric Rating Scale, *OSBD* Observational Scale of Behavioral Distress, *OSBD-r* Observational Scale of Behavioral Distress-revised, *PD* Passive distraction, *PP* Procedural preparation, *PSI-SF* Parenting Stress Index Short Form, *PSS* Perceived Stress Scale, *SC* Standard care, *STAI-CH* Spielberger State-Trait Anxiety Inventory for Children, *VAS* Visual Analogue Scale, *VAS-A* Visual Analog Scale-Anxiety, *VGD* Video game distraction, *VR* Virtual reality, *YCPC* Young Child PTSD Checklist

**Table 4:** Key results of included studies

Reference	Outcomes	Results
**Procedural preparation and distraction**		
Brown [44]	Pain	*• FPS-R* scores lower in Ditto™ than control at post-application of 2nd COD (MD = -1.51 [CI:-2.89, − 0.13] *p = 0.032*). *• HR* lowered across 3 CODs in Ditto™ group (MD = -4.89 [CI:-9.69, − 0.09], *p = 0.046*).
	Pain and distress	*• FLACC* scores not reported.
	Anxiety	*• VAS-A* scores lower in Ditto™ than control at pre-removal (MD = -1.79 [CI:-3.59, −0.01] *p* = 0.510).
	Trauma	*•* Intervention group did not affect *CTSQ* scores 1 week post-injury (MD not reported [CI:-1.49, 0.87] *p* = 0.602) or 3 months post re-epithelialisation (MD not reported [CI: −1.26, 2.00] *p* = 0.651).
Burns-Nader [45]	Pain	*•* Intervention group did not affect *FACES* scores (*p* = 0.290). *• Nurse’s pain reports* lower in tablet group (M = 3.73, SD = 0.88) than control (M = 2.93, SD = 1.03), (*p = 0.030*).
	Anxiety	*• CEMS* scores higher in control during (*p = 0.001*) and after (*p = 0.002*) hydrotherapy. *•* CEMS scores remained higher in control post-procedure (*p < 0.050*), tablet group returned to baseline levels (*p* = 0.570).
Miller^a^ [53]	Pain	*• FACES* scores sig. differed at pre and post-removal, and pre and post-application of all 3 CODs (*p* = ≤0.001 all time points). *• FACES* scores lowered across 3 COD’s in: ◦ MMD-D at pre-removal (*p* = ≤0.001), post-removal (*p* = 0.005), and pre-application (*p* = 0.004). ◦ MMD-PP at pre-removal (*p* = 0.044). ◦ VGD at post-application (*p* = 0.030). *• FACES* scores lowered in: ◦ MMD-PP more than VGD and control at pre-removal (both *p* = ≤0.010), post-removal (both *p* = < 0.001), pre-application (both *p* = < 0.001), and post-application (both *p* = < 0.001). ◦ MMD-D more than control at pre-application (*p* = ≤0.050), post-removal (*p* = < 0.001), and post-application (*p* = < 0.001); and VGD at post-removal (*p* = < 0.001) and post-application (*p* = < 0.001). *• VAS* scores sig. differed at pre-removal of 2nd and 3rd COD; and post-removal, pre and post-application of all 3 CODs (*p* = ≤0.001). *• VAS* scores increased across 3 CODs in VGD compared to MMD-PP at post-removal and application (both *p* = < 0.001), and MMD-D at post-removal (*p* = ≤0.050) and post-application (*p* = ≤0.001). *• VAS* scores lowered across 3 COD’s in: ◦ MMD-D at pre and post-removal, and pre-application (all *p* = ≤0.001), and post-application (*p* = 0.002). ◦ MMD-PP at pre-removal (*p* = 0.035), and post-application (*p* = 0.009). ◦ Control at pre-removal (*p* = 0.034). *•* VAS scores lowered in MMD-PP and MMD-D more than control at post-removal (both *p* = < 0.001) and post-application (both *p* = < 0.001).
	Pain and distress	*• FLACC* scores sig. differed at pre-removal of 2nd and 3rd COD (*p* = ≤0.001); post-removal at 1st (*p* = 0.003), 2nd, and 3rd CODs (*p* ≤ 0.001); pre-application of 1st (*p* = 0.010), 2nd, and 3rd CODs (*p* ≤ 0.001); and post-application of all 3 CODs (*p* ≤ 0.001). *• FLACC* scores lowered across 3 COD’s in: ◦ MMD-D at post-removal (*p* = 0.008), pre-application (*p* = 0.047), and post-application (*p* = 0.018). ◦ Control at pre-removal (*p* = ≤0.001). *•* FLACC scores lowered in: ◦ MMD-PP more than control at post-removal (*p* = ≤0.050) and post-application (*p* = < 0.001); and VGD at post-removal (*p* = ≤0.050) and post-application (*p* = ≤0.001). ◦ MMD-D more than control at post-removal (*p* = < 0.010), and post-application (*p* = < 0.001); and VGD at post-removal (*p* = ≤0.010) and post-application (*p* = < 0.001).
Miller^b^ [54]	Pain	*• FACES* scores lower in MMD than control at pre-removal *(p* = *0.004)*; post-removal, and pre and post-application *(all p* = *< 0.001)*, 30% reduction. *• VAS* scores lower in MMD than control at pre-removal (*p = 0.018*), post-removal (*p = 0.010*), pre-application (*p = 0.001*), and post-application (*p = < 0.001*), 30% reduction. *•* MMD combined sig. lowered pre-removal *FACES (p = 0.009)* and *VAS* scores *(p = 0.035)* compared to MMD-D. *• HR l*owered in MMD at removal and application *(*both *p = 0.040)*.
	Pain and distress	*• FLACC* scores lower in MMD than control at post-removal *(p = < 0.001)*, pre-application *(p = 0.021)*, and post-application *(p < 0.001)*, 50% reduction at removal. *•* MMD combined borderline less effective than MMD-D in reducing post-removal *FLACC* scores *(p = 0.050)*.
Jeffs [50]	Pain	*• APPT-WGRS* pre-procedure scores highest in VR, then SC and PD *(p = 0.041).* *• APPT-WGRS* procedure scores lower in VR than PD (MD = 23.70 mm [CI:2.40, 45.00] *p = 0.029*), and SC (MD = 9.70 mm [CI:-9.50, 28.90] not sig. *p* = 0.320). *•* Male patients reported less procedural pain (MD = 32.60 mm [CI: 14.90, 50.20] *p = <.001*).
	Anxiety	*•* Intervention group did not affect state (*p* = 0.060) or trait anxiety (*p* = 0.710).
Kipping [52]	Pain	*•* Intervention group did not affect patient *VAS* scores at dressing removal (*p* = 0.160) or application (*p* = 0.400). *•* Intervention group did not affect caregiver *VAS* scores at dressing removal (*p* = 0.710) or application (*p* = 0.750).
	Pain and distress	*• FLACC* scores lower in VR (M = 2.90, SD = 2.40) than control (M = 4.70, SD = 2.50) at dressing removal (*p = 0.020*), but not application (*p* = 0.230).
Van der Heijden [58]	Pain	*•* Intervention group did not affect *COMFORT-B* scores before or after intervention (SMD = 0.04 [CI:-0.30, 0.38] *p* = 0.990). *• FACES* scores lower in MT than SC (*p = 0.050*); relevant sample MT (*n* = 13), SC (*n* = 5).
	Distress	*•* Intervention group did not affect *OSBD-r* scores before or after intervention (SMD = 0.11 [CI:-0.23, 0.45] *p* = 0.530). *•* Intervention group did not affect *FPS-R* scores (*p* = 0.200).
Whitehead-Pleaux [59]	Pain	*•* Intervention group did not affect *FACES* scores before (*p* = 0.181), or after procedure (*p* = 0.345). *•* MD in *HR* from before to after procedure greatest in control (*p* = 0.003).
	Distress	*• NAPI* scores higher in MT than control during procedure (*p = 0.020*).
	Anxiety	*• Fear Thermometer* scores higher in MT than control before (*p* = 0.043), and during procedures (*p* = 0.002), but not after (*p* = 0.228).
**Hypnosis and guided imagery**		
Chester [46]	Pain	*•* Intervention group did not affect overall *FPS-R* scores before, during, or after any procedure (*p*= > 0.100). *•* FPS-R scores lower in patients < 8 years at 3rd COD (MD = 4.71 [CI: 0.33, 9.09] *p = 0.040*); relevant sample 3 per group. *• NRS* scores lower in hypnotherapy than control at pre-removal of 3rd COD (MD = -0.91 [CI:-1.62, − 0.20] *p = 0.010)*. *•* Intervention group did not affect *NRS* scores at any other time point across 3 CODs (*p*= > 0.200). *• HR* lower in hypnotherapy than SC at pre-removal (MD = -15.20 [CI:-27.20, − 3.20] *p = 0.010*) and post-application of 3rd COD (MD = -15.49 [CI:-28.25, − 2.53] *p = 0.020)*.
	Pain and distress	*• FLACC* scores not reported.
	Anxiety	*•* Patients > 8 years *VAS-A* scores lower in hypnotherapy than SC at pre-removal of 2nd COD (MD = -0.80 [CI:-1.50, − 0.10] *p = 0.030)*. *•* Caregiver *VAS-A* scores for patients < 8 years lower in hypnotherapy than SC at pre-removal of 2nd (MD = -1.37 [CI:-2.57, − 0.16] *p = 0.030)*, and 3rd CODs (MD = -2.07 [CI:-3.64, − 0.49] *p = 0.010*).
	Trauma	*•* Patient *CPSS* impairment severity scores lower in hypnotherapy than SC (MD = 0.46 [CI:-0.01, 0.92] *p = 0.050)*. *•* Caregiver *YCPC* symptom severity scores for children < 7 years higher in hypnotherapy than SC (MD = 0.75 [CI:0.05, 1.45] *p = 0.040)*.
Foertsch [48]	Pain and anxiety	*• FACES* and *VAS* scores not analysed due to patient difficulty in comprehending tools.
	Distress	*•* Intervention group did not affect *OSBD* scores between groups (F_1,9_ = 0.18, *p*= > 0.500), or across 4 CODs (exact F_3,18_ = 1.10, *p* = < 0.300). *•* Cry behaviors correlated with verbal resistance at 2nd (r [22]=0.77, *p = < 0.010*), 3rd (r [22]=0.56, *p = < 0.050*), and 4th CODs (r [22]=0.49, *p = < 0.050*); with emotional support at 1st (r [23]=0.58, *p = < 0.050)*, and 2nd CODs (r [22]=0.88, *p = < 0.010*); and with verbal pain at 1st (r [23]=0.52, *p = < 0.050*), and 2nd CODs (r [22]=0.83, *p = < 0.010)*. *•* Female patients displayed higher verbal resistance at baseline (t [21]= − 2.40, *p = 0.020)*; and cry behaviors at 2nd-4th COD (t [20]= − 2.26, *p = 0.030)*.
**Therapeutic approaches**		
Blakeney [43]	Anxiety/ distress	*• CBCL* anxious and depressed scores sig. lowered from pre-intervention to 1 year post-intervention in intervention group (t = − 2.50, *p = .017*) and control (t = − 2.40, *p = .026*); however not between groups (*p* = > 0.300).
Elliott [47]	Pain and distress	*•* Group comparisons not possible. *• BTDS* scores reduced in intervention group by *25–52% (mean = 36.7%)* from baseline to post-intervention. *• BTDS* scores consistently increased for intervention group in therapist absence. *•* Patient’s preferred: relaxation, emotive imagery, distraction, imagery of pleasant scenery, and earning tangible reinforcement for coping techniques.
Hyland [49]	Pain	*•* CLT group received fewer additional analgesic medication during procedure than SC (*n* = 6, 12% vs *n* = 9, 18%). *•* Average CHEOPS scores lower in CLT (Mdn = 5.30, IQR: 4.50–6.70) than SC (Mdn = 6.00, IQR: 5.40–7.60), (CI: 0.10, 1.20, *p = 0.020*). *•* Nursing staff observed higher pre-procedural pain in CLT than SC (Mdn = 1.00, IQR: 0.00–2.00 vs. Mdn = 0.50, IQR: 0.00–1.00). *•* Intervention group did not affect nursing staff observations of procedural pain (Mdn = 2.00 for both groups). *• FACES* scores not reported.
	Pain and anxiety	*•* CLT caregivers observed higher patient pre-procedural pain than SC caregivers (Mdn = 3.50, IQR: 0.00–4.00 vs. Mdn = 3.00, IQR: 0.00–5.00). *•* CLT caregivers observed lower patient procedural pain than SC caregivers (Mdn = 2.00, IQR: 0.00–4.00 vs. Mdn = 3.00, IQR: 1.00–7.00). *•* Intervention group had no affect on caregiver observations of patient pre-procedural anxiety (Mdn = 2.00, IQR: 1.00–5.00 vs. Mdn = 2.00, IQR: 0.00–5.00). *•* CLT caregivers observed less patient procedural anxiety than SC caregivers (Mdn = 3.00, IQR: 1.00–6.00 vs. Mdn = 4.30, IQR: 1.00–8.00).
	Anxiety	*•* Intervention group did not affect average CFS scores (CI: 0.00–0.20, *p* = 0.300). *•* CLT caregivers had higher anxiety than SC caregivers at pre-procedure (Mdn = 7.00, IQR: 5.00–8.00 vs. Mdn = 6.00, IQR: 4.00–8.00), and during procedure (Mdn = 5.00, IQR: 1.00–7.00 vs. Mdn = 3.50, IQR: 2.00–7.50). *•* Nursing staff observed higher patient pre-procedural anxiety in CLT than SC (Mdn = 2.00, IQR: 0.00–4.00 vs. Mdn = 1.50, IQR: 0.00–3.00). *•* Intervention group did not affect nursing staff observations of patient procedural anxiety (Mdn = 2.00 for both groups).
Sveen [57]	Post-traumatic stress	*• IES-R* scores lower in intervention than control at 6 weeks post-randomization (β = − 11.50 [SE:3.88] *p = 0.003*) and 3mths post-intervention (β = − 7.89 [SE:3.38] *p = 0.020*). *•* Intervention group did not affect *IES-R* scores at baseline or 12mths post-intervention. *•* Intervention group did not affect caregivers *PSI-SF* or *PSS* scores at any time point during CODs. *•* Caregivers perceived the intervention as informative and meaningful, but time consuming.
Preparation & ‘patient control’		
Kavanagh [51]	Pain	*•* Intervention group required less analgesic pain medication in 1st 2 weeks of hospitalisation (*p = < 0.010*). *•* Intervention group received more analgesic medication between CODs (*p = < 0.025*). *• Nurse reports* not reported.
	Anxiety	*•* Maladaptive symptoms higher in SC than intervention in 1st 2 weeks of hospitalisation (*p = 0.043*). *•* Anxiety levels higher in SC than intervention in 1st 2 weeks, not sig. (*p* = 0.135).
Moore [55]	Pain	*•* Intervention group did not affect *FPS* scores from baseline, during, or post-procedure (*p* = 0.717).
	Pain and distress	*• FLACC* scores lower in MP than SC during CODs (0.50 vs 2.00 respectively), not sig. (*p* = 0.165).
	Anxiety	*•* Intervention group did not affect caregivers state anxiety from baseline to post-procedure (*p* = 0.421).
Quay [56]	Anxiety	*•* Caregivers were able to rehearse treatments and share concerns about returning home. *• STAI-CH* scores decreased in intervention caregivers of patients with > 30% TBSA burns at discharge *(p = < 0.050)* and 1st follow-up *(p = < 0.050).* *•* Intervention group did not affect *STAI-CH* scores for any patient’s, or caregiver of patients with < 30% TBSA burns at discharge or 1st follow-up visit.

*APPT-WGRS* Adolescent Paediatric Pain Tool, Word Graphic Rating Scale, *BTDS* Burn-Treatment Distress Scale, *CBCL* Children’s Behavior Checklist, *CEMS* Children’s Emotional Manifestation Scale, *CFS* Children’s Fear Scale, *CHEOPS* Children’s Hospital of Eastern Ontario Pain Scale, *CI* 95% confidence interval, *CLT* Child Life Therapy, *COD* Change of dressing, *COMFORT-B* COMFORT-Behavioral scale, *CPSS* Child PTSD Symptom Scale, *CTSQ* Child Trauma Screening Questionnaire, *FACES* Wong-Baker FACES pain rating scale, *FLACC* Faces Legs Arms Cry Consolability, *FPS* Faces Pain Scale, *FPS-R* Faces Pain Scale-Revised, *HR* Heart rate, *IES-R* Impact of Event Scale-Revised, *IQR* Interquartile range, *M* Mean, *MD* Mean difference, *Mdn* Median, *MMD* Multi-modal Device, *MMD-D* Multi-modal Device-Distraction, *MMD-PP* Multi-modal Device-Procedural Preparation, *MP* Medical play, *MT* Music therapy, *NAPI* Nursing Assessment of Pain Index, *NRS* Numeric Rating Scale, *OSBD* Observational Scale of Behavioral Distress, *OSBD-r* Observational Scale of Behavioral Distress-revised, *PD* Passive distraction, *PSI-SF* Parenting Stress Index Short Form, *PSS* Perceived Stress Scale, *SC* Standard care, *SD* Standard deviation, *SMD* Standardised mean difference, *STAI-CH* Spielberger State-Trait Anxiety Inventory for Children, *TBSA* Total Body Surface Area, *VAS* Visual Analogue Scale, *VAS-A* Visual Analog Scale-Anxiety, *VGD* Video game distraction, *VR* Virtual reality, *YCPC* Young Child PTSD Checklist

#### Cultural components

None of the 17 included studies incorporated Australian Aboriginal cultural components as presented in the *Dance of Life’s* model of Australian Aboriginal people’s perspectives of health and wellbeing. Blakeney et al.’s social skills psychoeducation was the only study to include Native American children (*n* = 2, 3%); however, this study did not assess intervention effect by ethnicity or incorporate any cultural components. Therefore, its ability to meet the needs of First Nations people (respectfully used here in reference to Indigenous peoples globally) could not be ascertained [43]. Moore et al. acknowledged the lack of cultural diversity within their study on medical play; however, this was brief and specific to the inclusion of African American and Hispanic families [55]. Similarly, Chester et al.’s study on hypnotherapy briefly acknowledged the lack of representation of Aboriginal and/or Torres Strait Islander children; however, did not elaborate on any potential implications or outline the ethnic/cultural diversity of included children [46].

#### Pain

Distraction based interventions had variable effects on patient pain. The Multi-Modal Device (MMD) and Ditto™ devices reduced patient self-reported pain [44, 53, 54], caregiver observations of pain [53, 54], and nurse observations of pain and distress [53, 54] when the procedural preparation story “Bobby get’s a burn” and interactive distraction games were provided together [44, 54], and separately [53]. The benefits of MMD distraction increased with repeated use and was borderline more effective in reducing nurse observations of pain and distress behaviours when used alone than in combination with procedural preparation [53, 54]. However, less interactive video game distractions were found to reduce self-reported pain and increase caregiver observations of pain over time compared to Multi-Modal Device – Procedure Preparation (MMD-PP), and Multi-Modal Device – Distraction (MMD-D) [53]; while also reducing nurse observations of pain and distress, but not self-reported pain compared to standard distraction [45].

Three-dimensional virtual reality increased self-reported pre-procedural pain and reduced self-reported procedural pain more effectively than passive distractions [50]. While off-the-shelf virtual reality increased nursing staff’s observations of pain and distress behaviours, but had no effect on patients’ or caregivers’ reports of pain [52]. Music therapy reduced self-reported pain compared to standard care when provided immediately following CODs [58, 59]; however, did not affect patients’ self-reported pain when provided during CODs [58, 59]. Likewise, ‘medical play’ prior to COD commencement did not affect patients’ self-reported pain; however, did reduce nursing staff observations of pain and distress behaviour at insignificant levels [55]. In contrast, the use of Child Life Therapy (CLT) reduced patients’ pain as observed by caregivers and an independent assessor; and increased nursing staff’s observations of pre-procedural pain [49]. Similarly, hypnotherapy reduced pain levels at the third COD as self-reported by patients <8 years of age, and caregivers [46]. Stress management during CODs reduced self-reported pain and distress from baseline to post-intervention; however, increased similarly to the control group when the therapist was absent [47]. Further, patients that received increased ‘patient control’ and ‘predictability’ required less analgesic medication during the first two weeks of hospitalisations, but more in between CODs [51].

#### Distress

The MMD and Ditto™ devices’ procedural preparation and distraction reduced patient self-reported distress [44, 54], compared to standard care which increased self-reported distress [44]. Similarly, stress and pain management during CODs reduced patient self-reported distress, but only in the presence of a therapist [47]. In contrast, the use of familiar imagery did not reduce patients’ self-reported distress or investigators’ observations of distress behaviours [48]. Likewise, music therapy during and following CODs did not reduce nurse observations of distress [58, 59], but rather increased observations of distress when performed during COD procedures [59].

#### Anxiety

Preparation for dressing procedures or hospital discharge reduced patients’ and caregivers’ anxiety [51, 55, 56]. Similarly, increased ‘predictability’ and ‘patient control’ during CODs reduced patient’s anxiety during the first two weeks of hospitalisation; however, not significantly [51]. Hospital discharge preparation reduced anxiety among caregivers of children with burns affecting ≥30% of their TBSA, but did not impact other caregivers’ or patients’ state anxiety during CODs [56]. Blakeney et al. found that providing psychoeducational programs had similar effects as standard care in reducing patient’s self-reported anxiety/depression scores from pre-intervention to one year post-intervention [43]. Hypnotherapy lowered patients’ pre-removal anxiety as reported by patients aged >8 years at second COD, and caregivers for patients aged <8 years at second and third CODs [46]. Child Life Therapy (CLT) reduced caregivers’ observations of patients’ procedural anxiety; however increased both caregiver anxiety before and during procedures, and nurse observations of anxiety at pre-procedure [49]. The Ditto™ device lowered self-reported anxiety at pre-removal for patient’s > 8 years [44]. Likewise, tablet based electronic game distraction reduced anxiety during and after COD procedures compared to standard distraction [45]. In contrast, virtual reality did not reduce patient anxiety during CODs [44, 50, 59]; and music therapy provided during CODs increased self-reported anxiety before and during procedure [59].

#### Trauma

Few studies measured psychological trauma symptoms and those that did had mixed results [44, 46, 57]. Sveen et al.’s online self-help program reduced patient post-traumatic stress scores six weeks post-baseline and three months post-intervention; however symptoms returned to baseline levels 12 months post-intervention [57]. The online self-help program also did not reduce caregivers’ actual or perceived stress at any time [57]. Hypnotherapy significantly lowered patient self-reported trauma impairment severity compared to standard care; however, also increased caregiver’s observations of trauma symptoms in their children aged < 7 years at three months post-injury [46]. The Ditto™ device did not reduce children’s stress or trauma symptoms one week following injury or three months following wound healing [44].

### Risk of bias

**Fig. 2:**
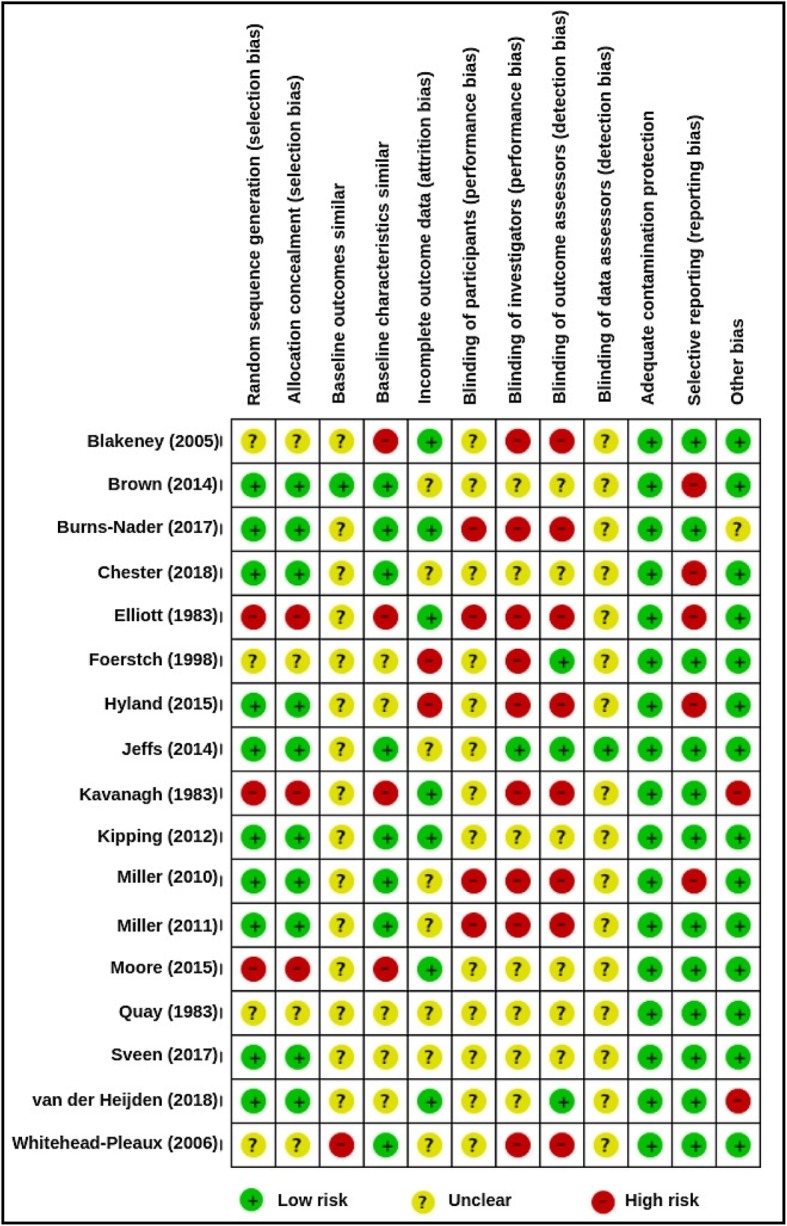
Risk of bias

Risk of bias assessment is presented in Fig. 2. Allocation blinding was often not possible for participants [45, 47, 50, 53, 54], investigators [43, 45, 47–49, 51, 53, 54, 59], and outcome or data assessors; however, only two studies attempted to reduce such bias with counter-rationales [47, 51]. Non-randomised control trials (NRCT) assigned participants based on attendance by month [51], weekday [55], or unspecified time [47]. Selective reporting potentially biased the impression of efficacy in some studies. This was present in Hyland et al. who did not present *Wong Baker FACES* pain scores [49]; Brown et al. and Chester et al. who did not report nurses’ *FLACC* measures [44, 46]; and Kavanagh who did not present *Nurse reports* of patient pain [51]. Missing data was acknowledged by Foertsch et al. [48] and Hyland et al. [49] but was not adequately addressed. Elliott & Olson reported minimal results with no group comparison due to heterogeneity in patient age, time of data collection, and length of hospitalisation [47].

Intervention and control groups differed at baseline. Blakeney et al.’s intervention group contained all (*n* = 2) Native American participants and more females than control [43]. Elliott & Olson’s ‘baseline’ participants were younger (M = 6.75 yrs) with greater burn TBSA (M = 32%) than intervention (Age M = 8.50 yrs. and TBSA% M = 21.50) [47]. Moore et al.’s groups significantly differed in burn location (*p* = 0.020) [55]. Kavanagh’s intervention group were all male, younger, with smaller burn TBSA, and shorter hospitalisation than control [51]. Whitehead-Pleaux et al.’s control had significantly lower baseline distress behaviours (*p* = 0.020) and self-reported anxiety (*p* = 0.040) than music therapy; however, the anxiety analysis included an unexplained extra participant [59].

### Downs & Black quality assessment

Studies were classified as excellent [46, 50, 52, 54], good [44, 45, 48, 49, 53, 55, 57, 58], fair [43, 56, 59], or poor quality [47, 51]. Three studies had small samples of six or less per group [47, 51, 59]. Some studies did not make clear if participants were recruited from the same population [43, 47], or over the same time period [43, 45, 47, 49, 59]; and only four studies adequately described adverse events [46, 50, 52, 54]. Two studies did not outline differences in follow-up lengths or perform adequate adjustments [47, 56]. Studies did not outline losses to follow-up [50, 51, 59]; or describe the characteristics of the source population [43–59], or those identified as lost to follow-up [43, 44, 47, 48, 53, 56, 57, 59]. Distribution of confounders were not outlined [47, 51, 56, 60], or clear imbalances inadequately addressed [43, 55]. Two studies performed seemingly unplanned analysis by burn TBSA [56] and patient age [58]; and data dredging were unclear for two studies [43, 51]. Two studies did not present random variability estimates [47, 51], and three did not present actual *p* values [47, 48, 56]. Furthermore, Kavanagh did not clearly describe outcome measures and stated some participants did not receive both aspects of the intervention [51].

## Discussion

This systematic review highlights a gap in understanding the effectiveness of psychosocial interventions for Aboriginal and Torres Strait Islander paediatric burn patients’ and their caregivers. Previous systematic reviews have not accounted for this specific group [60–64], assessed the effects of all psychosocial interventions available [60, 61], or considered psychological trauma as a primary outcome [60–63]. To the authors’ knowledge, this systematic review is the first to assess the effectiveness of psychosocial interventions in reducing pain and/or anxiety, distress, and trauma symptoms among paediatric burn patients and their caregivers as well as their relevance to Aboriginal and Torres Strait Islander peoples.

The appropriateness of the included interventions for Aboriginal and Torres Strait Islander people could not be determined due to the lack of cultural components and First Nations participants. The limited representation of First Nations people is disconcerting given all but one study [57] were conducted in countries with strong First Nations presence; including the United States of America (*n* = 9, 53%), Australia (*n* = 6, 35%), and South Africa (*n* = 1, 7%). The circumstance surrounding the omittance of First Nations people from most studies is unclear; however, some potential reasons may include inaccurate ethnicity records as exemplified by the categorisation of ‘lighter skin’ vs ‘darker skin’ by Brown et al. [44], or potential bias in intervention design appeal or accessibility for First Nations people.

Similarly, only two studies provided interventions to caregivers and focused on their symptoms [56, 57]. The limited focus on caregivers is also concerning given the strong evidence of psychological implications of paediatric burn injuries on caregivers [20, 23, 65]. Furthermore, sufficient psychosocial support, education and involvement of caregivers in CODs can build caregivers’ competency and aid with their coping during their child’s burn treatment [66, 67].

The relatively small number of included studies (*n* = 17) demonstrates the limited scope of formally assessed psychosocial interventions with clear comparison groups. We acknowledge the difficulty of applying rigour and standardisation to psychosocial interventions due to their need for flexibility and adaptability; attributes often key to their responsiveness to individual needs. Standardisation is further compounded by the multi-factorial nature of ‘standard’ burns care, resulting in varied COD approaches [44], length of time from pain medication to dressing removal [55], days of hospitalisation [56], informal procedural preparation [53], and ‘standard’ distraction during CODs [46]. However, some studies lacked standardisation of elements outside of ‘standard care’ that were provided to controls including additional investigator verbal support [48] and active distraction during CODs [51].

The generalisability of included studies was difficult to determine due to poor reporting of source population [43–59], and participants’ characteristics [47]. Small sample sizes of ≤20 per group impeded generalisability and power of ten studies [45, 47, 48, 50–55, 59]. Further, some studies excluded families involved with child protection services or prior Suspected Child Abuse and Neglect reports [44, 46, 52]; patients diagnosed with an impairment or Autism Spectrum Disorder [44]; and patients receiving initial CODs in theatre [46], requiring skin grafts, or other diagnosed medical conditions [44]. Other studies restricted inclusion by verbal communicability, inadvertently limiting representation of younger children [59] and non-English speaking families [44, 52]. And other studies demonstrated bias towards families with higher education [55, 57] and ‘socio-economic status’ [44], or with married/cohabitating caregivers and low family conflicts or symptoms of PTSD [57]. In contrast, Kavanagh’s intervention participants all reported a history of ‘family disorganisation’ or psychopathological symptoms, confounding factors to the intervention success [51]. And of particular concern, two included interventions were not tested among females due to gender imbalances between groups or lack of female participation [47, 51]. Potential gender differences in intervention experience or primary outcomes was not considered by any of the included studies.

This review was limited to studies available in English and with randomised control trial (RCT) and NRCT designs, resulting in the exclusion of seven otherwise relevant studies. These excluded studies were generally reflective of this review’s findings; however, three reported on interventions not captured here [68–70]. One study found art therapy effective in allowing paediatric burn patient’s to express their traumas [70], another found the combination of art and play therapy reduced patient anxiety [68], and the other found group therapy reduced caregiver anxiety [69]. It is highly recommended that the results of these excluded studies be considered when developing future interventions.

Authors of the included studies often criticised quantitative measures for being relatively subjective [49, 58] and heavily reliant on self-reports and structured assessments [58]. Studies reported the *Fear Thermometer* [59] and *Wong-Baker FACES scale* [48] to be confusing and difficult for young children to understand, despite being validated for use among children. Likewise, *FLACC behavioural pain scale* was heavily criticised as inappropriate for children aged 9+ years whom are less likely to display observable signs of distress and pain [48], and for its inability to capture pain and distress differences between groups [55]. Similarly, the *Achenbach Child Behavioral Checklist* [43], *State-Trait Anxiety Inventory* [56], and anxiety tool used by Kavanagh [51] were reportedly too general to accurately capture paediatric burn patients’ anxiety during CODs.

Despite these challenges, the included interventions demonstrated that procedural distraction via hand-held devices are effective in reducing patient’s pain [44, 45, 53, 54]; however, less effective when only games were available [45] opposed to procedural preparation stories as offered by the Ditto™ [44] and MMD devices [53, 54]. This indicates that procedural information provided at any time during procedures can reduce patient pain. This is supported by Damanhuri et al.’s finding that active distraction incorporating additional procedural information and encouragement was far more effective than passive distraction such as music therapy or games [14]. However, this review also found that distraction techniques did not reduce paediatric patient’s anxiety [44, 50, 59], or trauma symptoms [44] regardless of incorporation of procedural preparation. In contrast, therapeutic approaches were effective in reducing psychological morbidities among patients and caregivers. In particular, CLT reduced caregiver and independent assessor’s observations of patient pain, and caregiver’s observations of patient anxiety [49]. An online self-help program was the only intervention found to effectively reduce patient trauma symptoms [57]. Similarly, incorporating stress and pain management into CODs reduced patient distress but was not sustained in the therapist’s absence [47]. The results of the included studies should be interpreted with consideration of potential bias due to difficulties in intervention allocation blinding [43–59].

## Conclusion

This review returned a limited number of interventions that effectively reduced paediatric burn patient and caregiver psychological morbidities. The scarcity of work on reducing psychological trauma symptoms is particularly disconcerting given the volume of work emphasising the highly traumatising nature of burn injuries for both patients and families [2–4]. This highlights a need for additional work to better support and prepare caregivers for their vital role in providing security and comfort to their children during procedures. Of main concern to this review, the well-documented overrepresentation of Aboriginal and Torres Strait Islander paediatric burn patients was not reflected in the included studies nor were their perspectives on health and wellbeing. This lack of representation highlights the urgency for psychosocial interventions to be developed in partnership with and assessed among Aboriginal and Torres Strait Islander families. Finally, it is suggested that the effects of the included psychosocial interventions be further explored within broader healthcare settings and contexts; in particular, distraction featuring procedural information, CLT, stress and pain management, discharge preparation, and online self-help programs.

## Supplementary information

**Additional file 1.** Study search terms. Table outlining the search terms developed by the lead author in consultation with experts from the University of Queensland library.

**Additional file 2.** Data extraction points. Table outlining the topic areas and data points extracted from all applicable studies during the full text review stage.

**Additional file 3.** Data extraction form adapted from the *Dance of Life.* Table outlining the criteria for data point extraction developed in accordance with the *Dance of Life* and utilised on all applicable studies during the full text review.

## Data Availability

Not applicable.

## Abbreviations

ACT: Acceptance and Commitment Therapy
APPT-WGRS: Adolescent Paediatric Pain Tool, Word Graphic Rating Scale
BTDS: Burn-Treatment Distress Scale
C: Control
CBCL: Children’s Behavior Checklist
CBI: Children’s Behavior Inventory
CBT: Cognitive Behavioral Therapy
CEMS: Children’s Emotional Manifestation Scale
CFS: Children’s Fear Scale
CHEOPS: Children’s Hospital of Eastern Ontario Pain Scale
CI: 95% confidence interval
CINAHL: Cumulative Index to Nursing and Allied Health Literature
CLT: Child Life Therapy
COD: Change of dressing
COMFORT-B: COMFORT-Behavioral scale
CPSS: Child PTSD Symptom Scale
CTSQ: Child Trauma Screening Questionnaire
FACES: Wong-Baker FACES pain rating scale
FLACC: Faces Legs Arms Cry Consolability
FPS: Faces Pain Scale
FPS-R: Faces Pain Scale-Revised
HR: Heart rate
I: Intervention
IES-R: Impact of Event Scale-Revised
I^MMD-D^: Multi-modal Device-Distraction intervention
I^MMD-PP^: Multi-modal Device-Procedural Preparation intervention
I^PD^: Passive distraction intervention
IQR: Interquartile range
I^VGD^: Video game distraction intervention
I^VR^: Virtual reality intervention
M: Mean
MD: Mean difference
Mdn: Median
MMD: Multi-modal Device
MMD-D: Multi-modal Device-Distraction
MMD-PP: Multi-modal Device-Procedural Preparation
MP: Medical play
MT: Music therapy
NAPI: Nursing Assessment of Pain Index
NRCT: Non-randomised Control Trial
NRS: Numeric Rating Scale
OSBD: Observational Scale of Behavioral Distress
OSBD-r: Observational Scale of Behavioral Distress-revised
PD: Passive distraction
PP: Procedural preparation
PSI-SF: Parenting Stress Index Short Form
PSS: Perceived Stress Scale
PTSD: Post-traumatic Stress Disorder
RCT: Randomised Control Trial
SC: Standard care
SD: Standard deviation
SMD: Standardised mean difference
STAI-CH: Spielberger State-Trait Anxiety Inventory for Children
TBSA: Total Body Surface Area
VAS: Visual Analogue Scale
VAS-A: Visual Analog Scale-Anxiety
VGD: Video game distraction
VR: Virtual reality
YCPC: Young Child PTSD Checklist
