# Supplementary material for: Efficacy and cultural appropriateness of psychosocial interventions for paediatric burn patients and caregivers: a systematic review
Source: BMC Public Health. 2020 Mar 4;20:284. doi: 10.1186/s12889-020-8366-9 (PMC7057463; doi:10.1186/s12889-020-8366-9)
Supplement: Supplementary file 1 — Additional file 1. Study search terms. Table outlining the search terms developed by the lead author in consultation with experts from the University of Queensland library. [file 12889_2020_8366_MOESM1_ESM.docx]

# Additional file 1: Study search terms.

| **PICO** | **Search terms** |
| --- | --- |
| **Population** | 1. (Burns [MH] or burns) 2. (pediatrics [MH] or child [MH] or Infan* or babies or toddler* or minors or child* or adolescen* or youth or teen* or pediatric* or paediatric* or “young adult” or parent* or guardian*) |
| **Intervention** | 1. (psychology [MH] or "patient care team" [MH] or “Patient-Centered Care” [MH] or “Psychotherapy” [MH] or "support groups" or reintegration or "social skills" or “physical play” or "music therapy" or CBT or cognitive-behavioural or wellness or meditation or mindfulness or "peer support" or psych* or counsel* or “social work” or "support services" or "liaison officer" or yarning or therap* or distract* or hypno*) |
| **Outcomes** | 1. (“Depression” [MH] or “Psychological Stress” [MH] or “Panic Disorder” [MH] or “Mental Health” [MH] or “Post-traumatic stress disorders” [MH] or "Post-traumatic stress" or post-trauma* or trauma or PTSD or social-emotional or wellbeing or “well-being” or “mental health” or anxiety or depress* or stress or “quality of life” or QOL or “health related quality of life” or hrqol or healing or distress or pain*) |
