# Supplementary material for: Efficacy and cultural appropriateness of psychosocial interventions for paediatric burn patients and caregivers: a systematic review
Source: BMC Public Health. 2020 Mar 4;20:284. doi: 10.1186/s12889-020-8366-9 (PMC7057463; doi:10.1186/s12889-020-8366-9)
Supplement: Supplementary file 2 — Additional file 2. Data extraction points. Table outlining the topic areas and data points extracted from all applicable studies during the full text review stage. [file 12889_2020_8366_MOESM2_ESM.docx]

# Additional file 2: Data extraction points

| **Study Category** | **Data extracted** |
| --- | --- |
| **Study Design** | Intervention and study aims, cultural context, duration. |
| **Methods** | Recruitment processes, inclusion/exclusion criteria for participants, intervention groups, sample size, randomisation process, unit of analysis, appropriateness of statistical analysis. |
| **Recruitment and allocation** | Recruitment totals, % completed, % received allocated intervention, treatment of missing data. |
| **Participant characteristics** | Age, sex, race/ethnicity, education level, socio-economic status, literacy levels, first language, residence/demographic area, other socioeconomic demographics |
| **Intervention conditions** | Settings, theories, content, delivery, duration |
| **Outcome measure** | Psychometric properties, appropriateness to study type and target population, consistency among subgroups, translation to participants first language |
