# Supplementary material for: Efficacy and cultural appropriateness of psychosocial interventions for paediatric burn patients and caregivers: a systematic review
Source: BMC Public Health. 2020 Mar 4;20:284. doi: 10.1186/s12889-020-8366-9 (PMC7057463; doi:10.1186/s12889-020-8366-9)
Supplement: Supplementary file 3 — Additional file 3. Data extraction form adapted from the Dance of Life. Table outlining the criteria for data point extraction developed in accordance with the Dance of Life and utilised on all applicable studies during the full text review. [file 12889_2020_8366_MOESM3_ESM.docx]

# Additional file 3: Data extraction form adapted from the *Dance of Life.*

|  | **Present (1)/**  **Not present (0)** | **Details** | **Page/**  **Para #** |
| --- | --- | --- | --- |
| **Physical health and wellbeing** |  |  |  |
| *Study discusses…* |  |  |  |
| 1. Connection to country |  |  |  |
| 1. Traditional medicine |  |  |  |
| 1. Traditional diet and activity |  |  |  |
| 1. Negative physical health outcomes of colonisation |  |  |  |
| **Psychological health and wellbeing** |  |  |  |
| *Study discusses…* |  |  |  |
| 1. Sense of self, identify, autonomy, relatedness, or role within community |  |  |  |
| 1. Negative psychological health outcomes of colonisation |  |  |  |
| Intervention incorporates culturally valid tools, appropriate outcomes, accountability measures, cultural and spiritual phenomenology |  |  |  |
| **Social health and wellbeing** |  |  |  |
| *Study discusses…* |  |  |  |
| 1. Kinship systems, collectiveness, or community centred ideas |  |  |  |
| 1. Participant obligations and reciprocity to community |  |  |  |
| 1. Negative social health outcomes of colonisation including systematic barriers to culturally safe care |  |  |  |
| Intervention incorporates whole of life concepts, narrative therapies, or emphasises empowerment |  |  |  |
| **Spiritual health and wellbeing** |  |  |  |
| *Study discusses…* |  |  |  |
| 1. Dreamtime philosophies and beliefs |  |  |  |
| 1. Importance of belonging and connectivity to country |  |  |  |
| 1. Values of wisdom |  |  |  |
| **Cultural health and wellbeing** |  |  |  |
| *Study discusses…* |  |  |  |
| 1. Concepts of lore, language, ceremony, or healing beliefs |  |  |  |
| 1. Endurance, resilience, or strengths |  |  |  |
| Intervention incorporates concepts of lore, language, healing, and traditions |  |  |  |
